# National Pension Fund

**Published:** 1887-11-26

**Authors:** 


### THE NATIONAL PENSION FUND.

An Afternoon Conference.

**To meet the wishes of many interested in this Fund who reside away from London, it has been determined to hold an afternoon conference on Thursday, the 8th December, at the Society of Arts, John Street, Adelphi. Sir Andrew Clark, Bart., M.D., F.R.S., will take the chair at three p.m., and will be supported by the Countess of Strangford, Lady Clark, and others. Meanwhile, any who wish points to be cleared up, or who have found a difficulty in understanding the scheme, will facilitate its successful initiation by communicating them in writing to the Secretary of the Hospitals Association, Norfolk House, Norfolk Street, Strand, W.C., from whom tickets may be obtained on application.**

